# Epidemiological Characterization of the 2017 Dengue Outbreak in Zhejiang, China and Molecular Characterization of the Viruses

**DOI:** 10.3389/fcimb.2018.00216

**Published:** 2018-07-04

**Authors:** Hao Yan, Zheyuan Ding, Juying Yan, Wenwu Yao, Junhang Pan, Zhangnv Yang, Xiuyu Lou, Haiyan Mao, Junfen Lin, Jimin Sun, Juan Hou, Haocheng Wu, Chen Wu, Yanjun Zhang

**Affiliations:** ^1^Department of Microbiology, Zhejiang Provincial Center for Disease Control and Prevention, Hangzhou, China; ^2^Department of Public Health Surveillance and Advisory, Zhejiang Provincial Center for Disease Control and Prevention, Hangzhou, China; ^3^Department of Communicable Disease Control and Prevention, Zhejiang Provincial Center for Disease Control and Prevention, Hangzhou, China

**Keywords:** dengue fever, dengue virus, molecular characterization, E gene, phylogenetic analysis, epidemiological analysis, Zhejiang

## Abstract

Dengue, a mosquito-borne disease caused by the dengue virus (DV), has been recognized as a global public health threat. In 2017, an unexpected dengue outbreak occurred in Zhejiang, China. To clarify and characterize the causative agent of this outbreak, data on dengue fever cases were collected from the China Information System for Disease Control and Prevention in Zhejiang province for subsequent epidemiological analysis. A total of 1,229 cases were reported, including 1,149 indigenous and 80 imported cases. Most indigenous cases (1,128 cases) were in Hangzhou. The epidemic peak occurred in late August and early September, and the incidence rate of elderly people (4.34 per 100,000) was relatively high. Imported cases were reported all year round, and most were from South-East Asia and Western Pacific regions. Young people and men accounted for a large fraction of the cases. Acute phase serums of patients were collected for virus isolation. And 35 isolates (including 25 DV-2, 8 DV-1, 1 DV-3, and 1 DV-4) were obtained after inoculation and culture in mosquito C6/36 cells. The E genes of the 35 new DV isolates and the complete genome of a DV-2 isolate (Zhejiang/HZ33/2017), and the E gene of a DV-2 isolate from *Ae. albopictus* (Zhejiang/Aedes-1/2017) were determined. Phylogenetic analyses were performed using the neighbor-joining method with the Tajima-Nei model. Phylogenetically, DVs of all four serotypes with multiple genotypes (mainly including 21 Cosmopolitan genotype DV-2, 4 Asian I genotype DV-2, 6 genotype I DV-1, and 2 genotype V DV-1) were present in the indigenous and imported cases in Zhejiang during the same period. Most of the isolates probably originated from South-East Asia and Western Pacific countries. The imported cases, high density of mosquito vector, and missed diagnosis might contribute to the 2017 outbreak in Zhejiang.

## Introduction

Dengue is a mosquito-borne viral infection causing a severe flu-like illness and, sometimes causing a potentially lethal complication called severe dengue (previously known as dengue hemorrhagic fever). The vast distribution of mosquito vectors, which is fueled by frequent human migration, and a lack of adequate listed vaccines and effective drugs have resulted in a dramatic increase in dengue virus (DV) infections, which have undergone 30-fold growth over the past 50 years (Li et al., 2017). One recent estimate has indicated 390 million dengue infections per year, of which 96 million manifest clinically (Bhatt et al., 2013). Another study on the prevalence of dengue has estimated that 3.9 billion people in 128 countries are at risk of infection with DV (Brady et al., 2012). DV is a single-stranded, positive-sense RNA virus which belong to the genus *Flavivirus*, family *Flaviviridae*. Its genome is approximately 10,700 bp, comprising a single open reading frame that encodes three structural proteins (C, capsid; prM/M, precursor of membrane; and E, envelope) and seven non-structural proteins (NS1, NS2a, NS2b, NS3, NS4a, NS4b, and NS5) (Kuhn et al., 2002). DV can be grouped into four distinct serotypes (DV-1, DV-2, DV-3, and DV-4), each of which can be sub-classified into several genotypes on the basis of E gene sequences (Li et al., 2017).

Most countries in Asia have reported epidemic dengue outbreaks, including Myanmar, Laos, Thailand, Nepal, Malaysia, Indonesia, Cambodia, the Philippines, Vietnam, Japan, and Pakistan (Jarman et al., 2008; Holmes et al., 2009; Vu et al., 2010; Dubot-Pérès et al., 2013; Akram and Idrees, 2016; Ngwe Tun et al., 2016; Tajima et al., 2017). China has seen an increase in dengue fever (DF) cases in recent years. Since the first reported dengue outbreak in 1978, outbreaks have been reported in many provinces of mainland China, including Hainan, Guangxi, Guangdong, Yunnan, Fujian, and Zhejiang (Chen and Liu, 2015; Sun et al., 2017). Moreover, all four DV serotypes have been identified and found to be prevalent in China.

Zhejiang province, which has a typical subtropical climate, lies along the southeast coast of China. The topographic features in this province are complex, including plains, hills, and mountain lands, thus creating ideal conditions for the growth of mosquitoes (Guo et al., 2014). Zhejiang has a population of about 56.57 million. *Ae. albopictus* is the only vector for DV transmission in Zhejiang. In 2004, a DF outbreak caused by an indigenous patient who had traveled from Thailand occurred in Cixi, a city in the northeast of Zhejiang, and 83 cases were reported (Xu et al., 2007). A larger epidemic occurred in 2009, and there were 196 cases in Yiwu (Sun et al., 2011). In recent years, sporadic imported cases of DF have been reported almost annually in Zhejiang Province.

In 2017, an unexpected large dengue epidemic occurred, and a total of 1229 DF cases (93.8% emerging in Hangzhou) but no deaths were reported in Zhejiang, China. The DV cases in Zhejiang Province were mainly DV serotype 2, with a small fraction of DV serotype 1, 3, and 4. To analyze the molecular evolution of the virus, a phylogenetic tree can be constructed by using specific regions of the DV genome, such as the full-length sequence, E, or E/NS1 (Huang et al., 2012; Wang et al., 2016). In this study, we analyzed the phylogenetic, molecular, and epidemiological characteristics of DV outbreaks, investigated the origin of the 36 new dengue isolates and reported the complete genome sequence of a DV-2 isolate (Zhejiang/HZ33/2017) isolated from the 2017 outbreak in Zhejiang.

## Materials and methods

### Data sources

DF case data for the year 2017 were acquired from the National Notifiable Infectious Disease Reporting Information System (NNIDRIS) at the China Information System for Disease Control and Prevention in Zhejiang province. In China, DF is classified as a class-B notifiable infectious disease. Cases of DF must be reported to NNIDRIS within 24 h after diagnosis. Dengue cases were diagnosed according to the criteria for DF from the Chinese Ministry of Health. Information on each dengue case included demographic characteristics, laboratory confirmation, onset data, diagnosis data, and whether the case was imported or indigenous. An imported dengue case was defined as an infected person whose whereabouts could be traced to an origin in a dengue-endemic area outside Zhejiang Province within 15 days of the onset data; otherwise, cases were defined as indigenous. Characteristics of dengue cases were described by age, sex, occupation composition, and temporal and spatial distribution. Incidence was calculated as the number of cases divided by the population size during the study period. Temporal and spatial analyses of indigenous and imported cases were performed in Zhejiang Province, and spatiotemporal analysis of indigenous cases was performed in 13 districts of Hangzhou. Statistical analyses were performed by using ArcGIS 10.3 (ESRI, Redlands, CA, USA) and R statistical software, version 3.2.2 (R Development Core Team 2015).

### Vector monitoring

The mosquitoes were collected in sweeping nets at various communities located in the main urban area of Hangzhou during the 2017 dengue outbreak. The pooled mosquitoes were then stored at −80°C until virus detection. DV nucleotide detection, serotype identification and E gene amplification used the methods that mentioned below. Breteau index (BI, number of positive containers per 100 houses) was used to record *Ae. albopictus* infestation levels. Vector surveillance continued until BI was less than 5 according to dengue guidelines for diagnosis, treatment, prevention and control of the World Health Organization.

### Sample collection and laboratory diagnosis

A total of 90 serum samples of patients were obtained from the municipal Centers for Disease Control and Prevention (CDC) of Zhejiang. DV nucleotide detection and serotype identification were performed according to diagnostic criteria for DF (WS 216–2008) of the Ministry of Health of China.

### Virus isolation

Patients' positive acute phase serums (collected within 1–5 days of illness onset) were inoculated in *Ae. albopictus* mosquito C6/36 cells, which were a gift from the National Institute for Viral Disease Control and Prevention. The C6/36 cell line was cultured in minimum essential medium (MEM) (Gibco, USA) supplemented with 2% fetal bovine serum (Gibco, USA) at 28°C in 5% CO_2_. When complete cytopathic effects (CPE) were observed, culture supernatant was collected and stored at −80°C until use.

### RNA extraction and genome sequencing

Viral RNA was extracted with a RNeasy Mini Kit (Qiagen, Germany) according to the manufacturer's instructions. Complete genome sequencing of the DV-2 isolate from an indigenous case in Hangzhou (Zhejiang/HZ33/2017) was performed as previously described (Zhao et al., 2014). The E genes of the other 35 DVs were amplified with the following primers: DV1-E-F, 5′CAAGAACCGAAACRTGGATGTC3′ and DV1-E-R, 5′GGCTGATCGAATTCCACACAC3′; DV2-E-F, 5′AACATGGATGTCATCAGAAGG3′ and DV2-E-R, 5′CCAATCTTGTTACTGAGCGG3′; DV3-E-F, 5′GCCCTATTTCTTGCCCATTACA3′ and DV3-E-R, 5′CCGCACACTCCATTCTCCCAA3′ (Aquino et al., 2006); DV4-E/777F, 5′GCTTGGAAACATGCTCAGAG3′, DV4-E/1766R, 5′ACATGTGGTTTCCATCACCG3′, DV4-E/1639F, 5′TGGTGACATTCAAGGTTCCTC3′, and DV4-E/2509R, 5′ACTGTTCTGTCCAAGTGTGC3′ (Cao-Lormeau et al., 2011). Reverse transcription-polymerase chain reaction (RT-PCR) was carried out in one step with the following protocol: initial reverse transcription at 50°C for 30 min; denaturation at 94°C for 2 min; 40 cycles of denaturation at 94°C for 30 s, annealing at 53°C for 30 s, and extension at 72 °C for 2 min; and a final extension step at 72 °C for 10 min. Agarose gel electrophoresis (1.5%) was used to analyze the PCR products, which were then sequenced by a commercial facility (Tsingke Biotechnology Ltd, Hangzhou, China).

### Sequence alignment and phylogenetic analysis

The sequence was spliced in SEQMAN from the LaserGene package (DNASTAR Inc., Madison, WI). The nucleotide sequences were aligned using the Clustal W multiple sequence alignment program. DV reference sequences were downloaded from the GenBank database. The phylogenetic analysis based on full genome and E gene sequences was carried out by using the neighbor-joining method with the Tajima-Nei model in MEGA version 6.06 (http://www.megasoftware.net/). Bootstrap values ≥70%, calculated from 1,000 replicates, are shown at the tree branches. DV genotype was analyzed by including related reference sequences with known genotypes in the phylogenetic tree.

### Ethics statement

This study was approved by the Institutional Ethical Committee of Zhejiang Provincial Center for Disease Control and Prevention. Written informed consent was obtained from each participant.

## Results

### Epidemiological characteristics

#### Indigenous cases

Since July 2017, a dengue outbreak has attacked Zhejiang Province, China. The index case was a 28-year-old man with fever, headache, fatigue, rash, myalgia, and arthralgia began on August 14 and diagnosed on August 22. A total of 1,149 indigenous cases were reported, including 962 confirmed cases and 187 clinically diagnosed cases. There were 573 infected men and 576 infected women (0.99:1 ratio), and the incidence rate was 2.00 and 2.11 per 100,000, respectively. The median age was 51 (IQR: 35–63). The incidence rate of elderly people was relatively high, and retirees accounted for nearly one third of all cases (Table 1).

**Table 1:** Demographic characteristics of dengue cases in Zhejiang Province, 2017.

	**Indigenous cases**		**Imported cases**	
	**Number**	**Incidence rate/10^5^**	**N**	**Incidence rate/10^5^**
Total	1,149	2.07	80	0.14
Sex				
Male	573	2.00	53	0.19
Female	576	2.11	27	0.10
Age (median, IQR)	51,35–63		35,27.5–50.5	
< 20	48	0.45	1	0.01
20~	302	1.66	49	0.27
40~	399	2.22	23	0.13
60~	348	4.47	7	0.09
80~	52	3.63	0	0.00
Occupation		(Constituent ratio %)		
Retiree	346	30.11	5	6.25
Commercial service people	159	13.84	15	18.75
Household/Unemployed person	150	13.05	3	3.75
Industrial worker	139	12.10	14	17.5
Cadres	130	11.31	13	16.25
Farmer	50	4.35	15	18.75
Other	175	15.23	15	18.75

**Figure 1:**
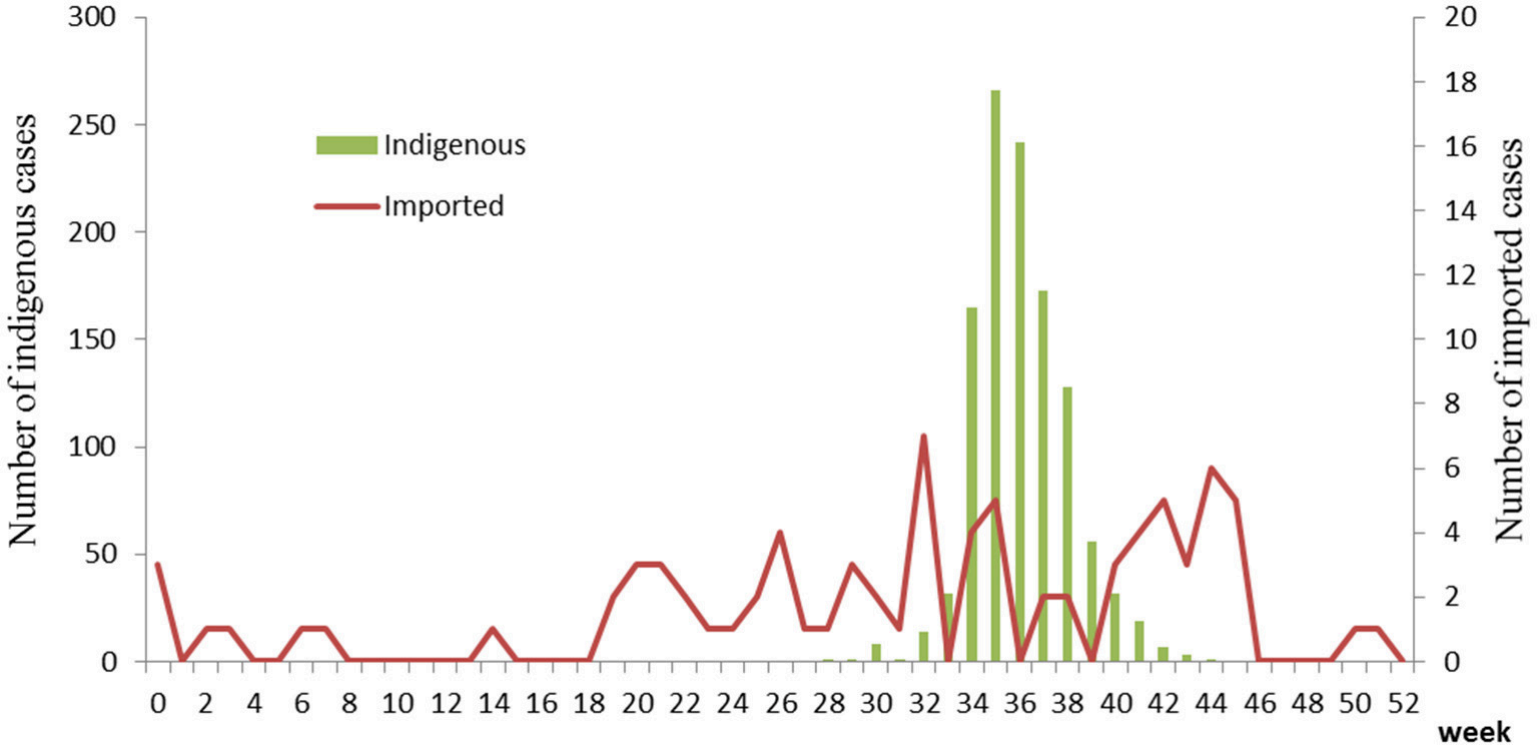
Temporal distribution of dengue cases in Zhejiang, China, 2017.

**Figure 2:**
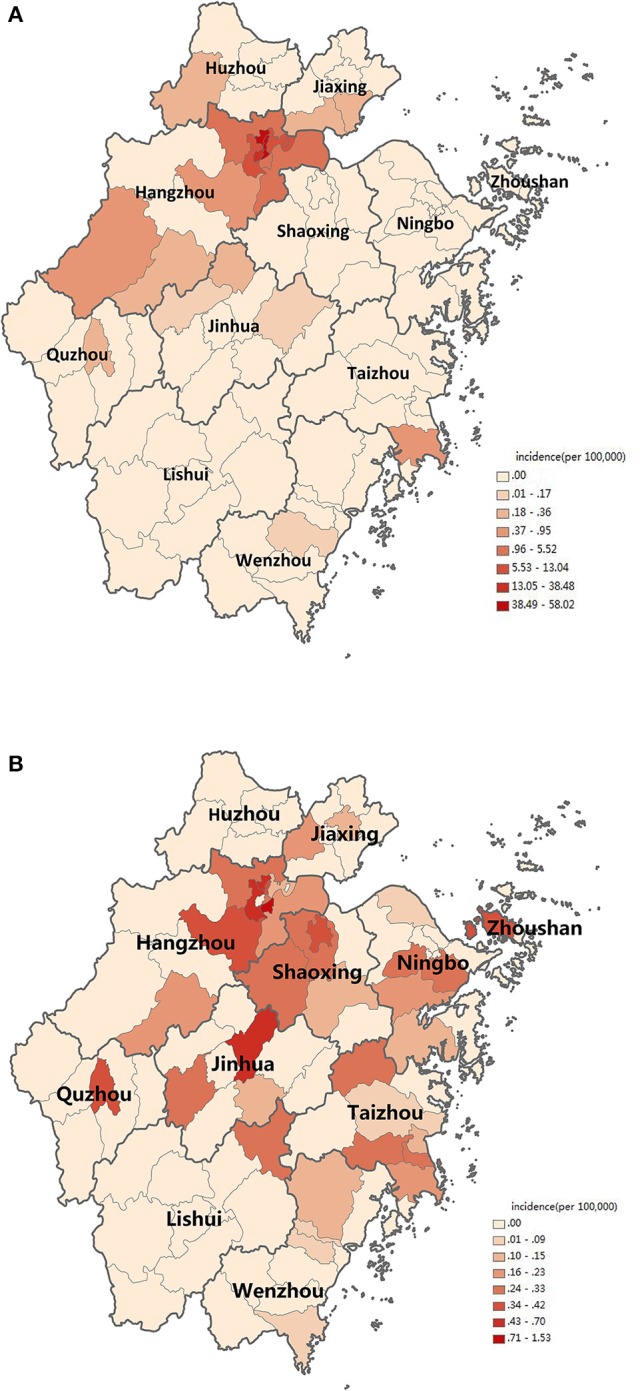
Distribution of dengue cases in Zhejiang, China, 2017. **(A)** Distribution of indigenous dengue cases. **(B)** Distribution of imported dengue cases. Incidence is marked on the map according to counties in Zhejiang Province.

**Figure 3:**
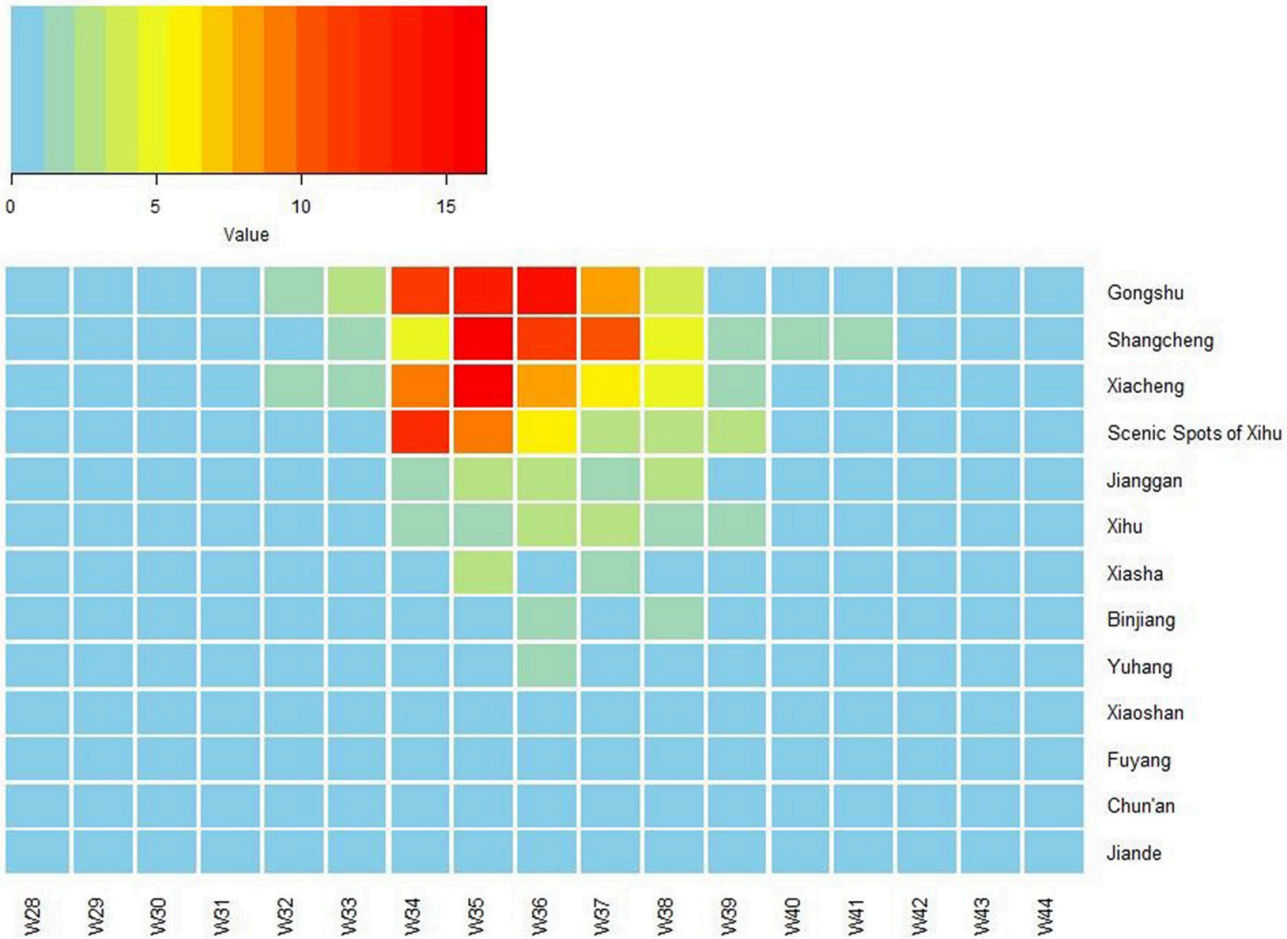
Heat map of incidence of imported dengue cases by 13 counties of Hangzhou, Zhejiang Province, China, 2017. The X-axis lists week number in 2017, and the Y-axis lists the name of 13 counties of Hangzhou. W, week.

The first case began on July 15. The number of cases increased markedly from 34th week, reached a peak in 35–36th week, and then began to decrease (Figure 1). The highest daily number of cases was 53 on August 28. There were 21 cases living outside of Hangzhou, which were distributed among the six prefecture-level cities Taizhou, Jiaxing, Jinhua, Wenzhou, Huzhou and Quzhou, which had 11, 4, 3, 1, 1, and 1 cases, respectively. The remaining 1,128 cases were in Hangzhou, the provincial capital of Zhejiang. All counties except for Tonglu and Lin'an in Hangzhou reported indigenous dengue cases. Three main urban areas of Hangzhou (Gongshu, Xiacheng, and Shangcheng) were the most hard-hit areas, consisting of more than 70% cases. The epidemic radiated out from these areas but was limited in Hangzhou and its surrounding regions (Figure 2A). Spatiotemporal analysis showed that the epidemic started in Gongshu and Xiacheng district, followed by neighboring districts (Shangcheng, Jianggan, and Xihu), and then Yuhang, Xiaoshan, and Xiasha (Hangzhou Economic and Technological Development Area). The epidemic was most severe in Gongshu, Xiacheng, and Shangcheng during 34th to 37th week (Figure 3).

#### Imported cases

A total of 80 imported dengue cases were reported in Zhejiang Province during the study period, including 53 men and 27 women (1.96:1 ratio). The median age was 35 (IQR: 27.5–50.5), and the incidences in the age groups of 20–39 years old were relatively high. Farmer, commercial service people, industrial worker and cadres accounted for most cases (Table 1).

Imported cases were reported year round. Around 32–35th and 42–45th week, the case number increased slightly (Figure 1). Most cases distributed in the north region of Zhejiang Province (Figure 2B). Most cases were imported from South-East Asia (42 cases) and Western Pacific (27 cases) regions, in which the top countries was Thailand (20 cases) and Vietnam (11 cases), respectively (Table 2).

**Table 2:** Distribution of countries of origin for imported dengue cases.

**Original country**	**Number of cases**
South-East Asia Region	42
Thailand	20
Myanmar	7
India	6
Sri Lanka	5
Indonesia	2
Maldives	1
Bangladesh	1
Western Pacific Region	27
Viet Nam	11
Cambodia	5
Philippines	5
Laos	3
Malaysia	3
African Region	7
Tanzania	2
Cote d'Ivoire	2
Angola	1
Ethiopia	1
Mauritius	1
Other Provinces in China	4
Total	80

### Entomologic investigation

A total of 136 mosquitoes were collected in a community in Shangcheng district of Hangzhou, of which 52 were identified based on morphology as *Ae. albopictus*, whereas 84 were identified as *Culex*. They were divided into one pool respectively. The *Culex* pool was negative for DV genome, while the *Ae. albopictus* pool was positive for DV-2. The BIs of the main urban area of Hangzhou were extremely high in the first statistics in 35th week (Gongshu 16, Xiacheng 27, Xihu 40.33, and Shangcheng 23.30). With the process of mosquito eradication, the BIs decreased rapidly and were all less than 5 in 37th week (Gongshu 0, Xiacheng 2.6, Xihu 4.53, and Shangcheng 2.3).

### Laboratory diagnosis, virus isolation, and genome sequencing

Eighty-one of the 90 collected serum samples were DV RNA positive, including 55 indigenous cases and 26 imported cases. Serotype identification results indicated the presence of all four serotypes DVs in Zhejiang Province in 2017: 68 DV-2 cases (50 indigenous and 18 imported), 10 DV-1 cases (5 indigenous and 5 imported), 2 DV-3 imported cases, and one DV-4 imported case. Among 43 randomly-selected acute phase serums that were positive for DV RNA, 35 DV isolates were successfully obtained after inoculation and culture in C6/36 cells. Routine RT-PCR was performed with virus culture and the supernatant of the pooled *Ae. albopictus* mosquitoes homogenates to amplify the whole genome and E gene. The complete genome sequences of DV-2 isolate Zhejiang/HZ33/2017 and the E gene of 35 additional isolates were obtained and submitted to the NCBI GenBank database (GenBank accession numbers: MH010595-MH010630).

### Phylogenetic analysis

Phylogenetic analysis was performed based on the sequences obtained, including reference sequences from the NCBI GenBank database. This analysis indicated that DVs of all four serotypes were present in indigenous and/or imported cases in Zhejiang Province, 2017 (Table 3).

**Table 3:** Serotype and genotype identification and amplification of the E gene of dengue cases in Zhejiang, China, 2017.

**Prefecture**	**Indigenous**	**Imported**	**Total**
Hangzhou	6	0	6
	II(6 with 6 Cosmopolitan genotype)^a^		II(6)
Taizhou	4	4	8
	I(4 with 2 genotype I and 2 genotype V)	I(1 with 1 genotype I) II(3 with 2 Cosmopolitan genotype and 1 Asian I genotype)	I(5) II(3)
Jiaxing	1	1	2
	II(1 with 1 Cosmopolitan genotype)	I(1 with 1 genotype I)	I(1) II(1)
Jinhua	3	3	6
	II(3 with 3 Cosmopolitan genotype)	II(2 with 2 Cosmopolitan genotype) III(1 with 1 genotype III)	II(5) III(1)
Huzhou	1	0	1
	II(1 with 1 Cosmopolitan genotype)		II(1)
Shaoxing	1	8	9
	II(1 with 1 Cosmopolitan genotype)	I(2 with 2 genotype I) II(6 with 3 Cosmopolitan genotype and 3 Asian I genotype)	I(2) II(7)
Yiwu	0	1	1
		II(1 with 1 Cosmopolitan genotype)	II(1)
Zhoushan	0	2	2
		II(1 with 1 Cosmopolitan genotype) IV(1 with 1 genotype I)	II(1) IV(1)

a *I, II, III, and IV is the 4 distinct serotypes: DV-1, DV-2, DV-3, and DV-4, respectively; the number in the bracket is the number of DV serotype and genotype*.

**Figure 4:**
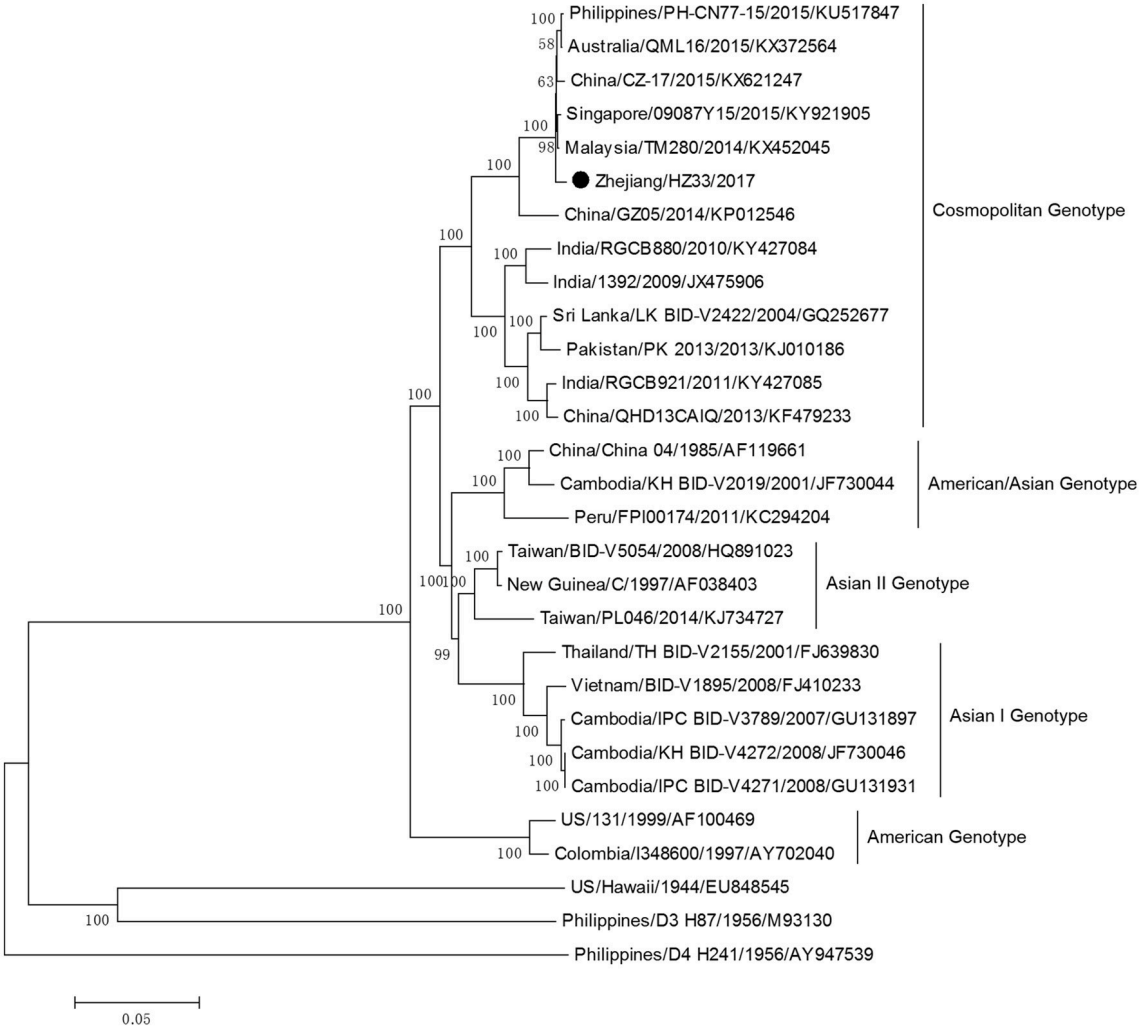
Phylogenetic tree constructed from full-length DV sequences from different countries. Zhejiang/HZ33/2017 isolated in this study is labeled with a black circle. DV-1 strain Hawaii, DV-3 strain H87 and DV-4 strain H241 were used as outgroups.

**Figure 5:**
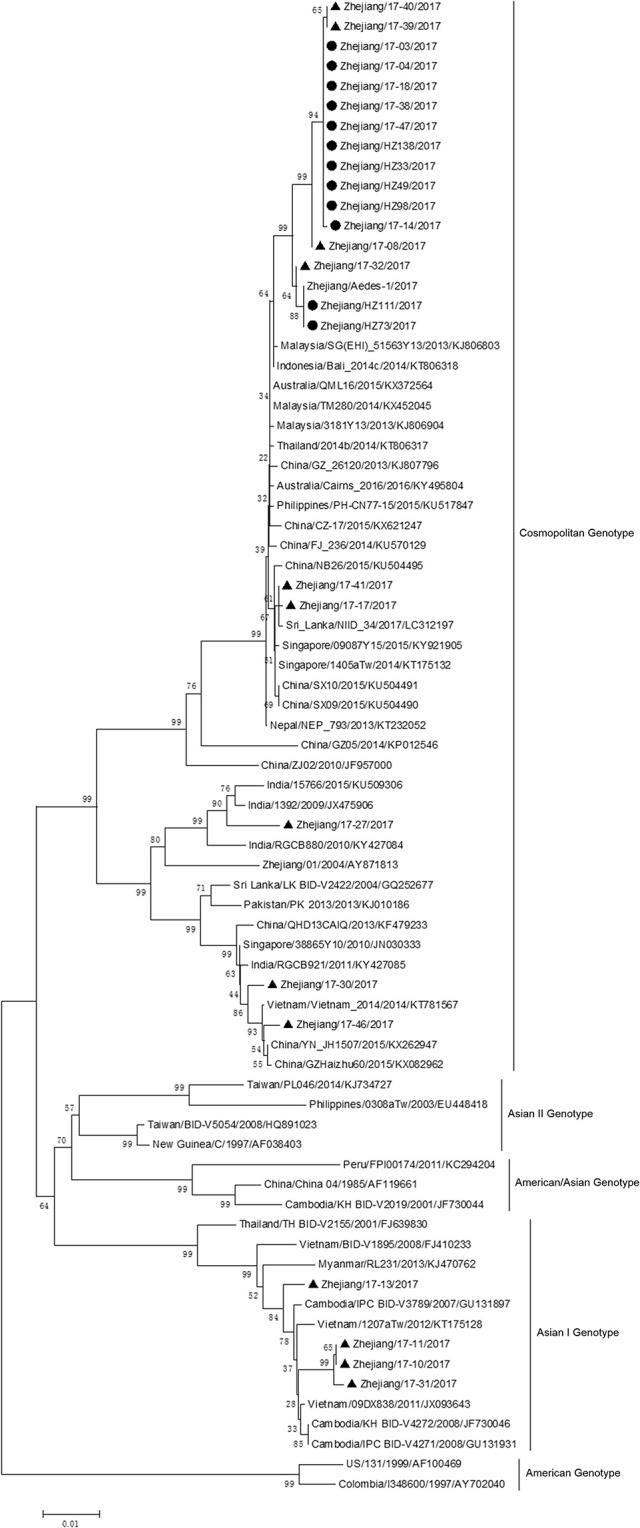
Phylogenetic analysis of DV-2 sequences based on the E gene. The phylogenetic tree was generated in MEGA version 6.06 using the neighbor-joining method with 1,000 bootstrap replicates. Isolates from indigenous cases are labeled with black circles, and isolates from imported cases are labeled with black triangles.

The complete genome sequences of isolate Zhejiang/HZ33/2017 and sequences from representative isolates of different countries were chosen to construct a phylogenetic tree, and sequences of the DV-1 strain Hawaii, DV-3 strain H87, and DV-4 strain H241 were used as outgroups. The phylogenetic reconstruction classified all the DV-2 isolates into five genotypes, and Zhejiang/HZ33/2017 was identified as the Cosmopolitan genotype and was closely related to viruses from Malaysia and Singapore (Figure 4). In recent years, the sequences of DV that have been uploaded mostly contain the E gene but fewer full-length genome. To further clarify the origin of Zhejiang/HZ33/2017, the 26 DV-2 isolates (including Zhejiang/HZ33/2017 and Zhejiang/Aedes-1/2017) generated from the E gene were aligned with reference isolates of various genotypes (Figure 5). The results showed that 22 isolates clustered into DV-2 Cosmopolitan genotype, in which all 12 isolates (Zhejiang/HZ33/2017, Zhejiang/HZ49/2017, Zhejiang/HZ73/2017, Zhejiang/HZ98/2017, Zhejiang/HZ111/2017, Zhejiang/HZ138/2017, Zhejiang/17-03/2017, Zhejiang/17-04/2017, Zhejiang/17-14/2017, Zhejiang/17-18/2017, Zhejiang/17-38/2017, and Zhejiang/17-47/2017) from indigenous cases, 4 isolates (Zhejiang/17-08/2017, Zhejiang/17-32/2017, Zhejiang/17-39/2017, and Zhejiang/17-40/2017) from imported cases, and Zhejiang/Aedes-1/2017 formed a tight subclade. The other 4 isolates from imported cases belonged to DV-2 Asian I genotype.

**Figure 6:**
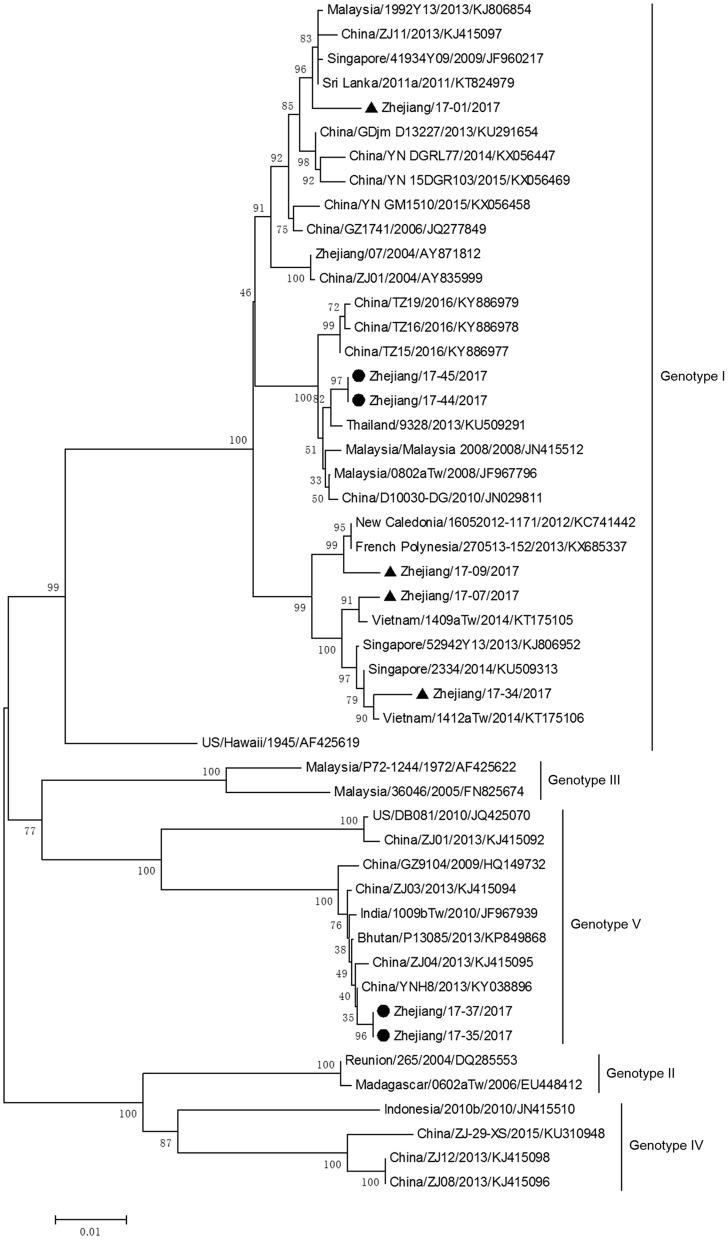
Phylogenetic analysis of DV-1 sequences based on the E gene. The phylogenetic tree was generated in MEGA version 6.06 using the neighbor-joining method with 1,000 bootstrap replicates. Isolates from indigenous cases are labeled with black circles, and isolates from imported cases are labeled with black triangles.

The phylogenetic tree based on E gene categorized all DV-1 isolates into five genotypes and revealed that DV-1 from Zhejiang Province had a different genotype (Figure 6). Two pairs of isolates from 4 indigenous cases in Taizhou city (located in the southeast of Zhejiang) belonged to DV-1 genotypes I and V, respectively. The first two isolates, Zhejiang/17-35/2017 and Zhejiang/17-37/2017, were closely related to viruses from Yunnan and Zhejiang, China (2013). The second two isolates, Zhejiang/17-44/2017 and Zhejiang/17-45/2017, clustered with virus from Thailand (2013).

**Figure 7:**
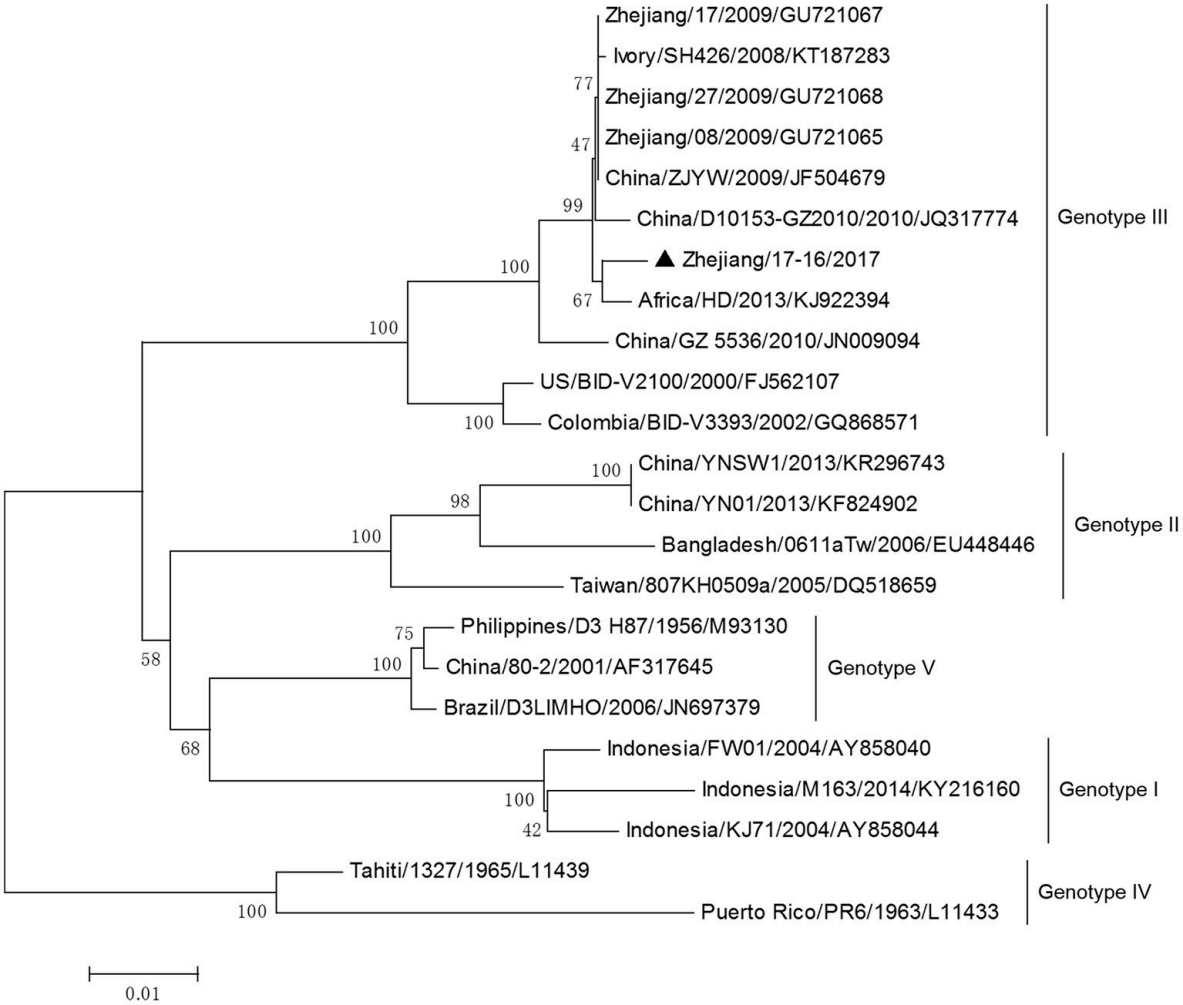
Phylogenetic analysis of DV-3 sequences based on the E gene. The phylogenetic tree was generated in MEGA version 6.06 using the neighbor-joining method with 1,000 bootstrap replicates. Isolates from imported cases are labeled with a black triangle.

**Figure 8:**
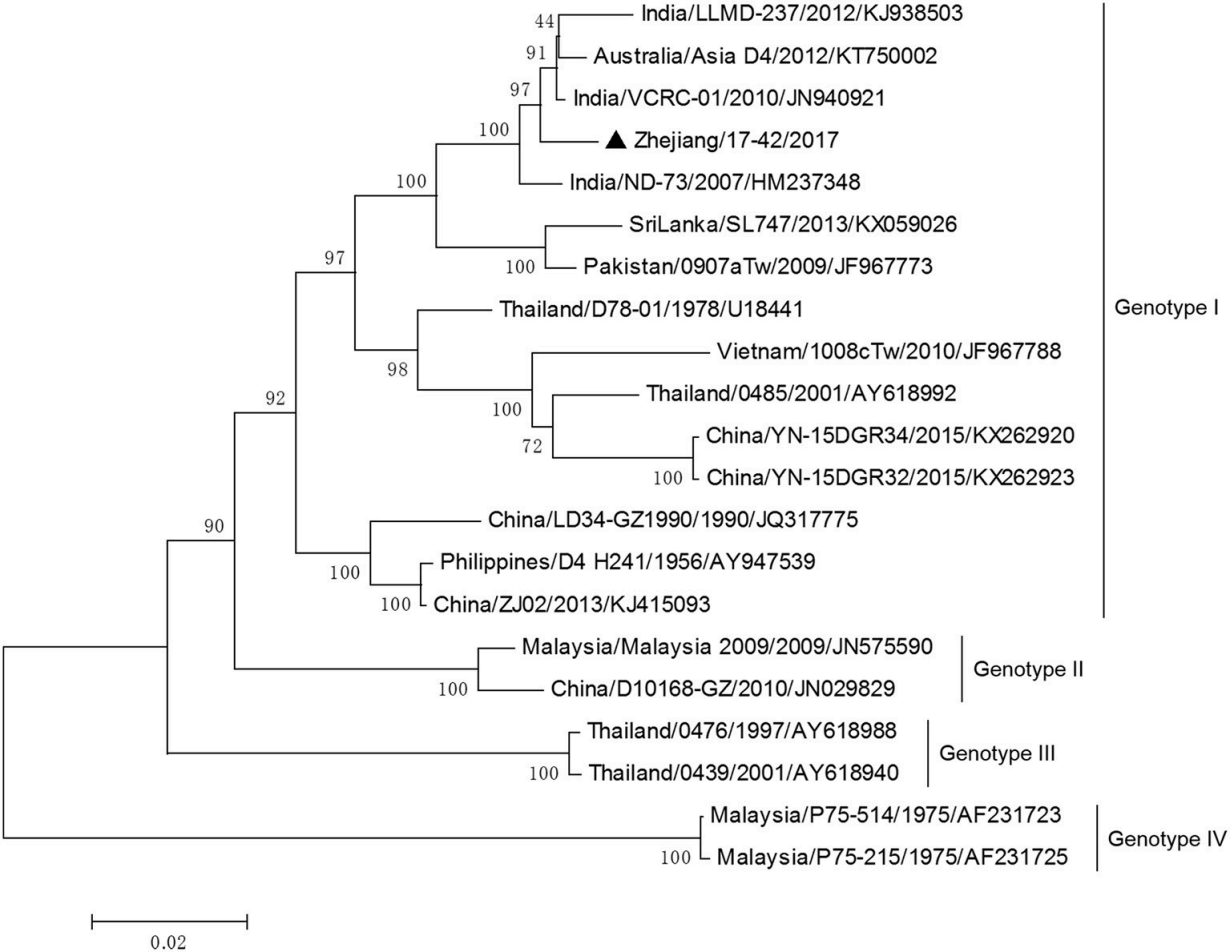
Phylogenetic analysis of DV-4 sequences based on the E gene. The phylogenetic tree was generated in MEGA version 6.06 using the neighbor-joining method with 1,000 bootstrap replicates. Isolates from imported cases are labeled with a black triangle.

DV-3 and DV-4 isolated from DF cases in Zhejiang in 2017 were all imported. Phylogenetic analysis indicated that the DV-3 isolate Zhejiang/17-16/2017 belonged to genotype III, clustering closely with virus from Africa (2013) (Figure 7). The isolate Zhejiang/17-42/2017 was identified as D-4 genotype I, closest relative to the isolates from India and Sri Lanka (Figure 8).

## Discussion

DF was clearly reported in the coastal provinces of China in the early twentieth century; and the first DF outbreak was identified in Guangdong province in 1978. Since the 1990s, dengue epidemics have spread gradually from Guangdong, Hainan, and Guangxi provinces in the southern coastal regions to the relatively northern and western regions including Fujian, Zhejiang, and Yunnan provinces. (Wu et al., 2010; Li et al., 2017). Since then, sporadic cases of DF have been reported almost every year in Zhejiang Province, and outbreaks occurred in the years 2004 and 2009 (Xu et al., 2007; Sun et al., 2011). This study was conducted to molecular and epidemiological characterize and phylogenetic analyze isolates from Zhejiang Province, in 2017. This local dengue outbreak was the largest and most severe in Zhejiang Province in the past 10 years. There are four possible reasons that may explain this outbreak. First, rapid development of globalization has caused global spread of DF worldwide, and the incidence of this disease has increased markedly both globally (Bhatt et al., 2013; Guzman and Harris, 2015) and in China (Ren et al., 2015). In Zhejiang Province, increasing tourism, trade, and investments with dengue-epidemic countries has led to high population mobility, which in turn has increased the risk of disease spread. Second, weather factors such as temperature and rainfall in summer-autumn season in Zhejiang are suitable for the survival and breeding of mosquito vectors. Third, dengue is not an epidemic disease in Zhejiang Province, so susceptibility in the population is high. Fourth, the rate of asymptomatic dengue infection is high (Wang et al., 2015), but the clinical diagnostic capacity is insufficient (Ding et al., 2018), so rapid and accurate diagnosis has been limited to a certain extent. Delay of diagnosis of imported dengue cases may increase the risk of transmission and local spread of dengue.

Elderly people had higher incidence than younger people in this outbreak, a finding consistent with those from studies in other areas of China, such as Guangdong (Xiao et al., 2016) and Taiwan (Lin et al., 2012), which have experienced local dengue epidemic outbreaks. This observation might be attributed to more time spent outdoors and a higher likelihood of existing chronic diseases in elderly people and retirees (Xiao et al., 2016). No significant difference in incidence between sexes was found, since both men and women were susceptible to DV. This outbreak first occurred in Gongshu district, an urban area in Hangzhou that has a high population density and mobility, and then spread to the neighboring districts and then the outskirts, thus indicating that the epidemic distribution spread over time. The most serious duration of the epidemic was in late August and early September, when the density of mosquitoes was high in Zhejiang Province (Wu et al., 2015). In the 35th week, high BIs were observed in the main urban area of Hangzhou, which was consistent with the peak reported. Because of the latent phase of dengue, the decline in new cases is lagging behind when the BIs reduce to less than 5 in 37th week. Once new foci were founded, anti-mosquito measures were carried out immediately by community health departments. As a result, only 136 mosquitoes were captured and only one positive *Ae. albopictus* pool was determined. And the virus infection situation in local mosquito vectors was still unclear.

Compared with indigenous cases, imported cases were mainly young people, and there were more men than women. The purposes of overseas travel were mainly tourism, then trade and labor export (Ding et al., 2018). Most of the countries of origin for these cases were in South-East Asia and Western Pacific, possibly because of the highly developed tourism industries in these regions, especially in Thailand and Vietnam. Cases were reported year round, thus leading to persistent risks of virus transmission. When weather conditions are suitable, and the density of mosquito vectors reaches a high level, local epidemics are prone to outbreak.

The phylogenetic tree of DV-2 based on full-length sequences indicated that Zhejiang/HZ33/2017 probably originated from Guangdong or a Southeast Asian country (Figure 4). The E protein of DV encoded by the E gene is responsible for cell receptor binding, which is the main target of neutralizing antibodies and vaccine development (Whitehead et al., 2007; Webster et al., 2009). In recent years, most DV sequences in GenBank were from the E gene only. To further determine the origin of Zhejiang/HZ33/2017 and the rest of the 25 new DV-2 isolates, a phylogenetic analysis based on the E gene was conducted (Figure 5). We found that the 26 new DV-2 isolates from Zhejiang belonged to Cosmopolitan genotype and Asian I genotype. All 12 isolates (Zhejiang/HZ33/2017, Zhejiang/HZ49/2017, Zhejiang/HZ73/2017, Zhejiang/HZ98/2017, Zhejiang/HZ111/2017, Zhejiang/HZ138/2017, Zhejiang/17-03/2017, Zhejiang/17-04/2017, Zhejiang/17-14/2017, Zhejiang/17-18/2017, Zhejiang/17-38/2017, and Zhejiang/17-47/2017) from indigenous cases, the Zhejiang/Aedes-1/2017, and 4 isolates (Zhejiang/17-40/2017, Zhejiang/17-39/2017, Zhejiang/17-08/2017, and Zhejiang/17-32/2017) from imported cases were clustered in the same subclade. We confirmed that the 6 indigenous cases (17-03, 17-04, 17-18, 17-38, 17-47, and 17-14) were imported from Hangzhou to other cities in Zhejiang. According to epidemiological data, 17-32, 17-08, 17-39, and 17-40 were imported from Malaysia and Thailand, so we speculated that HZ33, HZ49, HZ73, HZ98, HZ111, HZ138, and Aedes-1 were most likely originated from Malaysia or Thailand, rather than from Guangdong. Another 9 isolates (Zhejiang/17-41/2017, Zhejiang/17-17/2017, Zhejiang/17-27/2017, Zhejiang/17-30/2017, Zhejiang/17-46/2017, Zhejiang/17-13/2017, Zhejiang/17-11/2017, Zhejiang/17-10/2017, and Zhejiang/17-31/2017) from imported cases probably originated from South-East Asia or Western Pacific countries such as Sri Lanka, India, Vietnam, and Cambodia.

The rest of the 10 new isolates (8 DV-1, one DV-3, and one DV-4) were also analyzed based on their E gene sequences, respectively. Four DV-1 isolates from indigenous cases in Taizhou were located in two clusters belonging to genotypes I and V (Figure 6). Case 17-37 was a neighbor of 17–35, whose husband had returned from India before she became ill. The phylogenetic analysis showed that Zhejiang/17-35/2017 and Zhejiang/17-37/2017 were closely related to isolates from Yunnan and Zhejiang (KY038896 and KJ415095), but we further found that YNH8 (2013) originated from India, and ZJ04 (2013) was imported from Myanmar. After combining with epidemiological data, we supposed that Zhejiang/17-35/2017 and Zhejiang/17-37/2017 were likely originated from India. Zhejiang/17-44/2017 and Zhejiang/17-45/2017 (epidemiological information of the cases were limited) belonged to DV-1 genotype I and probably originated from Thailand. These four DV-1 isolates from indigenous cases had a certain phylogenetic distance to the viruses outbreak in Cixi, Zhejiang, 2004 (AY871812 and AY835999). The phylogenetic reconstruction indicated that the other 4 isolates (Zhejiang/17-01/2017, Zhejiang/17-09/2017, Zhejiang/17-07/2017, and Zhejiang/17-34/2017) from imported cases were likely originated from India, Vietnam, and Cambodia. The phylogenetic tree revealed that Zhejiang/17-16/2017, which was imported from Nigeria, had a close link with virus from Africa (2013). Moreover, Zhejiang/17-42/2017, which was imported from India, was closely related to the isolates from India and Sri Lanka, thus suggesting that they were most likely originated from those corresponding countries (Figures 7, 8).

This was a large outbreak with 1,229 cases, including 1,153 cases (1,128 indigenous and 25 imported) in Hangzhou, the provincial capital of Zhejiang. DV-2 was the dominant serotype epidemic in Zhejiang; however, all four serotypes emerged during the same period. Although Zhejiang does not belong to the endemic area of dengue, there are vector mosquitoes and environmental conditions supporting DV transmission in some regions of Zhejiang. There is always a risk of local outbreak caused by imported cases, such as the DV-1 and DV-3 outbreaks in 2004 and 2009, respectively. There are imported dengue cases in Hangzhou every year that do not cause a large scale endemic transmission every time. Hence, why was there a dengue outbreak in 2017? We inferred some probable reasons that may have led to this unexpected large outbreak. First, for Hangzhou residents, some South-East Asia and Western Pacific countries, including Thailand, Myanmar, Sri Lanka, Vietnam, Malaysia, are popular tourist destinations. Second, Hangzhou is a beautiful and economically advanced city in China that experiences high population mobility every year, as notably occurred after the Group 20 Summit in 2016. Third, long periods of high temperature and abundant precipitation in the summer and fall of 2017 in Hangzhou probably led to more frequent mosquito activity and facilitated mosquito breeding (Yang et al., 2009; Sarfraz et al., 2014). Fourth, DV infection often causes severe flu-like clinical symptoms that are easily misdiagnosed by inexperienced clinicians, thus leading to the occurrence of secondary cases or even the outbreak of dengue.

In our study, it was found that DVs of all four serotypes have been identified in Zhejiang Province. During 2017, the DV-2 Cosmopolitan genotype and DV-1 genotypes I and V were verified in indigenous cases. Multiple isolates of each serotype were present, and this may have increased patients' risk of dengue hemorrhagic fever and dengue shock syndrome, and threatened patients' lives (Morens, 1994; Gubler, 2011). Fortunately, DV-3 and DV-4 were detected only in sporadic imported cases. The present study reported the complete genome sequence of a clinical DV-2 isolate and investigated the origin of the 36 new DV isolates during this outbreak in Zhejiang Province. However, extensive virological studies and comprehensive epidemiological investigation are warranted in the future.

Our results indicated that imported DF patients from South-East Asia and Western Pacific countries were probably the primary cause of the DF epidemics in Zhejiang Province. Widely distributed of *Ae. albopictus* in urban areas increase the risk of indigenous dengue transmission and indigenous DF epidemics in Zhejiang Province. Because no effective vaccines and drugs are available for dengue, prevention and control measures should be strengthened. Determining the origin of the 2017 isolates from Zhejiang Province might provide information that could lead to more effective control measures. Efforts should be made in improving the capabilities of clinicians to diagnose this disease, improving and disseminating proper preventive measures in high-risk populations, surveillance of imported cases, strengthening vector control, and prevention and control of local epidemics.

## Author contributions

YZ designed and supervised the experiments. HY, JY, WY, JP, and XL carried out the experiments. HY, ZD, JY, JP, JS, HW, and CW analyzed the data. HY and ZD wrote the paper. JY, HY, JH, and HM performed sample collections. ZY, HM, and JL provided helpful suggestions about the study. All authors read and approved the final manuscript.

### Conflict of interest statement

The authors declare that the research was conducted in the absence of any commercial or financial relationships that could be construed as a potential conflict of interest.
